# Activity-dependent release of phosphorylated human tau from *Drosophila* neurons in primary culture

**DOI:** 10.1016/j.jbc.2021.101108

**Published:** 2021-08-30

**Authors:** Sazan Ismael, Ghadir Sindi, Robert A. Colvin, Daewoo Lee

**Affiliations:** Neuroscience Program, Department of Biological Sciences, and Molecular and Cellular Biology Interdisciplinary Graduate Program, Ohio University, Athens, Ohio, USA

**Keywords:** human tau release, phosphorylation, activity-dependent release, *Drosophila*, primary neuronal culture, AD, Alzheimer's disease, ATR, all-*trans* retinal, BL, blue light, CSF, cerebrospinal fluid, ChR2, channelrhodopsin 2, DIV, days *in vitro*, hTau, human tau, IP-CM, immunopurified conditioned media, LDH, lactate dehydrogenase, PHF, paired helical filament, PI, phosphatase inhibitor, PRD, proline-rich domain, pSite, phosphorylation site

## Abstract

Neuronal activity can enhance tau release and thus accelerate tauopathies. This activity-dependent tau release can be used to study the progression of tau pathology in Alzheimer's disease (AD), as hyperphosphorylated tau is implicated in AD pathogenesis and related tauopathies. However, our understanding of the mechanisms that regulate activity-dependent tau release from neurons and the role that tau phosphorylation plays in modulating activity-dependent tau release is still rudimentary. In this study, *Drosophila* neurons in primary culture expressing human tau (hTau) were used to study activity-dependent tau release. We found that hTau release was markedly increased by 50 mM KCl treatment for 1 h. A similar level of release was observed using optogenetic techniques, where genetically targeted neurons were stimulated for 30 min using blue light (470 nm). Our results showed that activity-dependent release of phosphoresistant hTau^S11A^ was reduced when compared with wildtype hTau. In contrast, release of phosphomimetic hTau^E14^ was increased upon activation. We found that released hTau was phosphorylated in its proline-rich and C-terminal domains using phosphorylation site-specific tau antibodies (*e.g.*, AT8). Fold changes in detectable levels of total or phosphorylated hTau in cell lysates or following immunopurification from conditioned media were consistent with preferential release of phosphorylated hTau after light stimulation. This study establishes an excellent model to investigate the mechanism of activity-dependent hTau release and to better understand the role of phosphorylated tau release in the pathogenesis of AD since it relates to alterations in the early stage of neurodegeneration associated with increased neuronal activity.

Intracellular neurofibrillary tangles are a pathological hallmark of Alzheimer's disease (AD) and other tauopathies (1). It is known that tangles are composed of microtubule-associated protein tau, which is hyperphosphorylated and aggregated (1, 2). Tau is not only an intracellular protein but also known to be released to the extracellular fluid (3, 4). Released tau can have both physiological and pathological roles in the nervous system (5, 6, 7). Interestingly, neuronal excitability increases during the early stages of AD (8, 9, 10, 11). For example, mild cognitive impairment in AD correlates with hyperactivity in the hippocampus (10, 12). Therefore, studies of activity-dependent release of tau on tauopathy are of significant interest in understanding how neuronal hyperexcitability and increased synaptic activity are involved in progression of AD (8, 9, 10, 11, 12, 13).

A handful of groups studied activity-dependent tau release (14, 15, 16) in addition to its spontaneous basal release. Pooler *et al.* (14) showed for the first time that tau can be released from rat cortical neurons in culture by glutamate agonists. Activity-dependent tau release was also observed *in vivo* (15). A recent study (16) used a sophisticated optogenetics approach to specifically activate a subset of neurons, which subsequently released tau. Neuronal activity appears to be an important factor regulating tau release, but its role in tau pathology is unknown. In addition, the mechanism of activity-dependent tau release is still elusive in terms of its secretion pathways (*e.g.*, membrane-free or vesicle/exosome-mediated tau release), species of released tau (*e.g.*, monomers, oligomers, or truncated) and function of released tau (*i.e.*, physiological *versus* pathological).

Tau undergoes several post-translational modifications, and phosphorylation is the most commonly observed (17, 18, 19). More than 40 phosphorylation sites (pSites) of tau are thought to be involved in AD pathogenesis (2, 4, 19). Phosphorylation of specific tau pSites is important for its association with microtubules and normal function, but its hyperphosphorylation is a common feature of tauopathies (17, 20). Indeed, some of these tau pSites are known to be abnormally phosphorylated in paired helical filaments (PHFs) and neurofibrillary tangles during progression of AD but are not phosphorylated in healthy brains (3). Tau phosphorylation is also known to increase its release from neurons (21, 22). Phosphorylated tau proteins at specific sites (*e.g.*, T181) were detected in cerebrospinal fluid (CSF) from patients with AD. However, there is still a significant gap in understanding how tau release is modulated by phosphorylation.

In this study, we developed a genetically tractable model to study activity-dependent tau release using *Drosophila* neurons in primary culture expressing human tau (hTau) and a variety of genetic approaches such as a binary UAS/Gal4 system (23) and optogenetics (24). We studied how tau phosphorylation contributes to its activity-dependent release using two tau mutant lines: phosphomimetic tau^E14^ (25) and phosphoresistant tau^S11A^ (26). Finally, a selected group of pSite-specific tau antibodies (*e.g.*, AT8) was used to examine the phosphorylation profiles of tau by released neuronal activity.

## Results

### Expression of hTau in *Drosophila* neuronal culture

**Figure 1:**
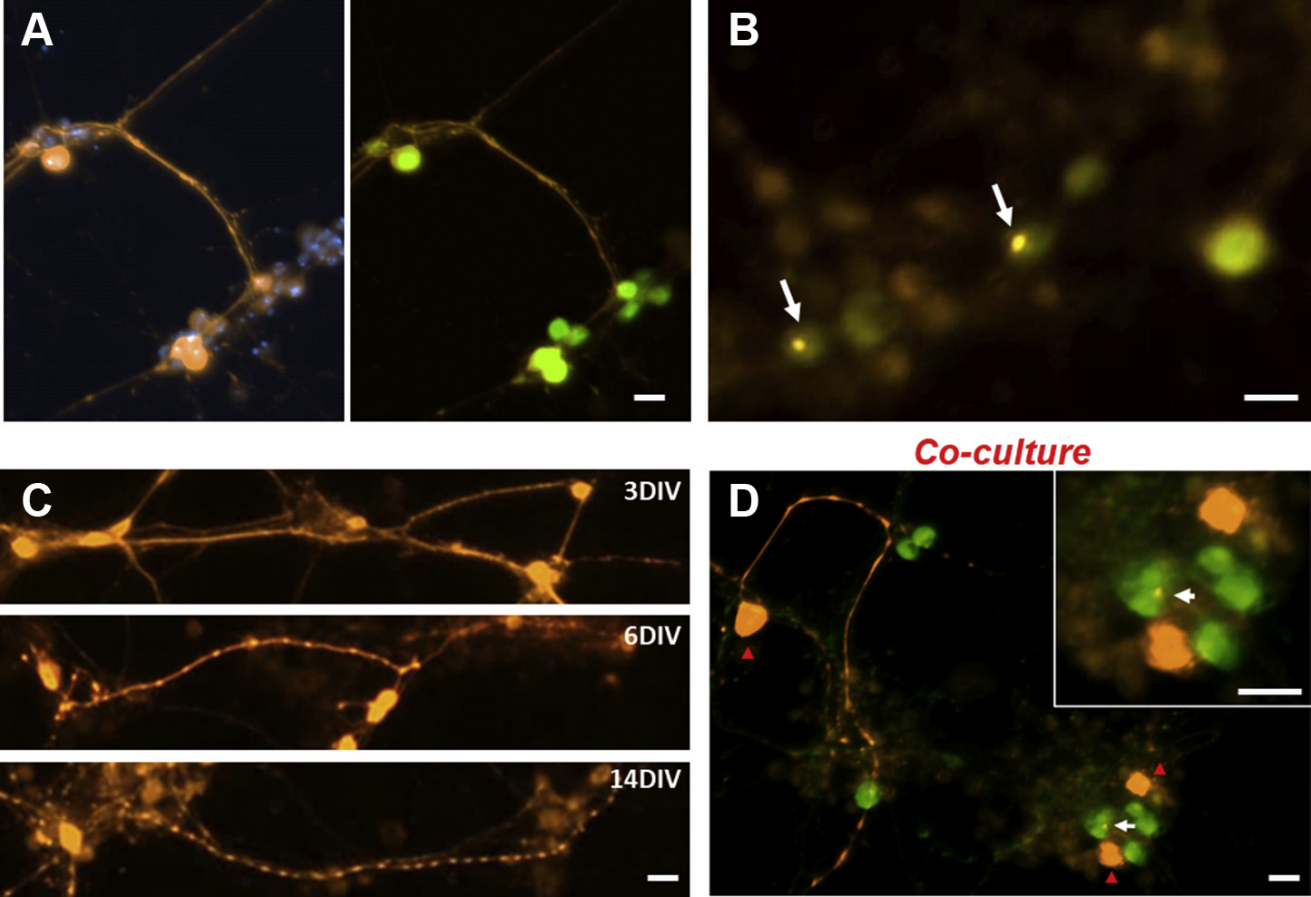
**Human tau (hTau) expressed in *Drosophila*-cultured neurons forms aggregates, causes neuritic degeneration, and induces its cell-to-cell propagation.***A*, hTau expression (*orange*) was observed in a subset of *Drosophila*-cultured neurons, not all neurons as DAPI (*blue*)-stained nuclei of all cells in culture. Cholinergic driver Cha-Gal4 was used to induce expression of hTau as well as GFP (Cha-Gal4, UAS-GFP × UAS-hTau). About 9 days *in vitro* (DIV) neurons were stained. *B*, aggregates of hTau were observed in a subset of neurons, which also expressed GFP (indicated by *arrows*). *C*, hTau was also expressed in the neuronal process and induced neurite fragmentation. This tendency was more noticeable in older neuronal cultures such as 6 or 14 DIV culture. *D*, cell-to-cell propagation of hTau in fly neuronal coculture. Coculture of primary neurons from two fly lines: Cha-Gal4 × UAS-hTau as a donor, Cha-Gal4 × UAS-GFP as a recipient line. In this field of view, three anti-hTau(+) neurons (*orange*, indicated by *red arrowheads*) are closely localized with GFP(+) recipient neurons. A small hTau aggregate (*bright yellow spot*, indicated by *white arrow*) was found in the soma of a recipient neuron labeled with GFP. *Inset*: enlarged image showing two donor neurons (*orange*) and four recipient neurons marked with GFP. The scale bar represents 10 μm. DAPI, 4′,6-diamidino-2-phenylindole.

In order to study activity-dependent release of hTau, we used *Drosophila* primary neuronal culture expressing hTau. Using Gal4 × UAS binary system (23), the fly line was made by crossing the cholinergic driver Cha-Gal4 with UAS-hTau^2N4R^ line. GFP transgene (UAS-GFP) was added to mark cholinergic neurons coexpressing hTau. The majority of GFP(+) neurons in ChaGFP-Gal4 × UAS-hTau^2N4R^ cultures at 9 days *in vitro* (DIV) was stained with antitotal hTau HT7 antibody (Fig. 1*A*) confirming the robust expression of hTau in GFP(+) neurons. We observed tau aggregates in a subset of cholinergic neurons (Fig. 1*B*). Furthermore, hTau-induced puncta formation and neurite fragmentation became more noticeable as neurons aged in culture (*e.g.*, 6 and 14 DIV; Fig. 1*C* and Table 1). These data suggest that expression of hTau in fly neuronal culture results in neurodegeneration and intracellular tau aggregation.

**Table 1:** Number of hTau punctae in neurites of cultured neurons at 3, 6, and 14 days DIV

DIV	Number of hTau punctae/100 μm	n
3	3.95 ± 0.69	14
6	9.44 ± 0.93^a^	17
14	17.73 ± 1.92^a^	19

Average numbers at 6 and 14 DIV were compared with that of three DIV for Student's *t* test. n = number of neurites used for analysis. Results were from three independent experiments.

a *p* < 0.001.

In the next experiment, we examined whether released extracellular hTau spread between neurons. To confirm hTau spreading, we cocultured two different fly lines: one expressing hTau (donor cells—Cha-Gal4 × UAS-hTau^2N4R^) and recipient cells expressing GFP without hTau expression (Cha-Gal4 × UAS-GFP). Embryonic neuroblast cells from the donor line were added to a coverslip, and then those from the recipient line were added later after the donor cells had settled to the bottom of the dish. hTau was detected in cholinergic GFP(+) recipient neurons by immunofluorescence suggesting cell-to-cell transfer of released hTau in *Drosophila* primary neuronal culture (Fig. 1*D*). Thus, this culture system will be useful to study mechanisms underlying tau release and cell-to-cell propagation.

### KCl-induced depolarization increases hTau release

**Figure 2:**
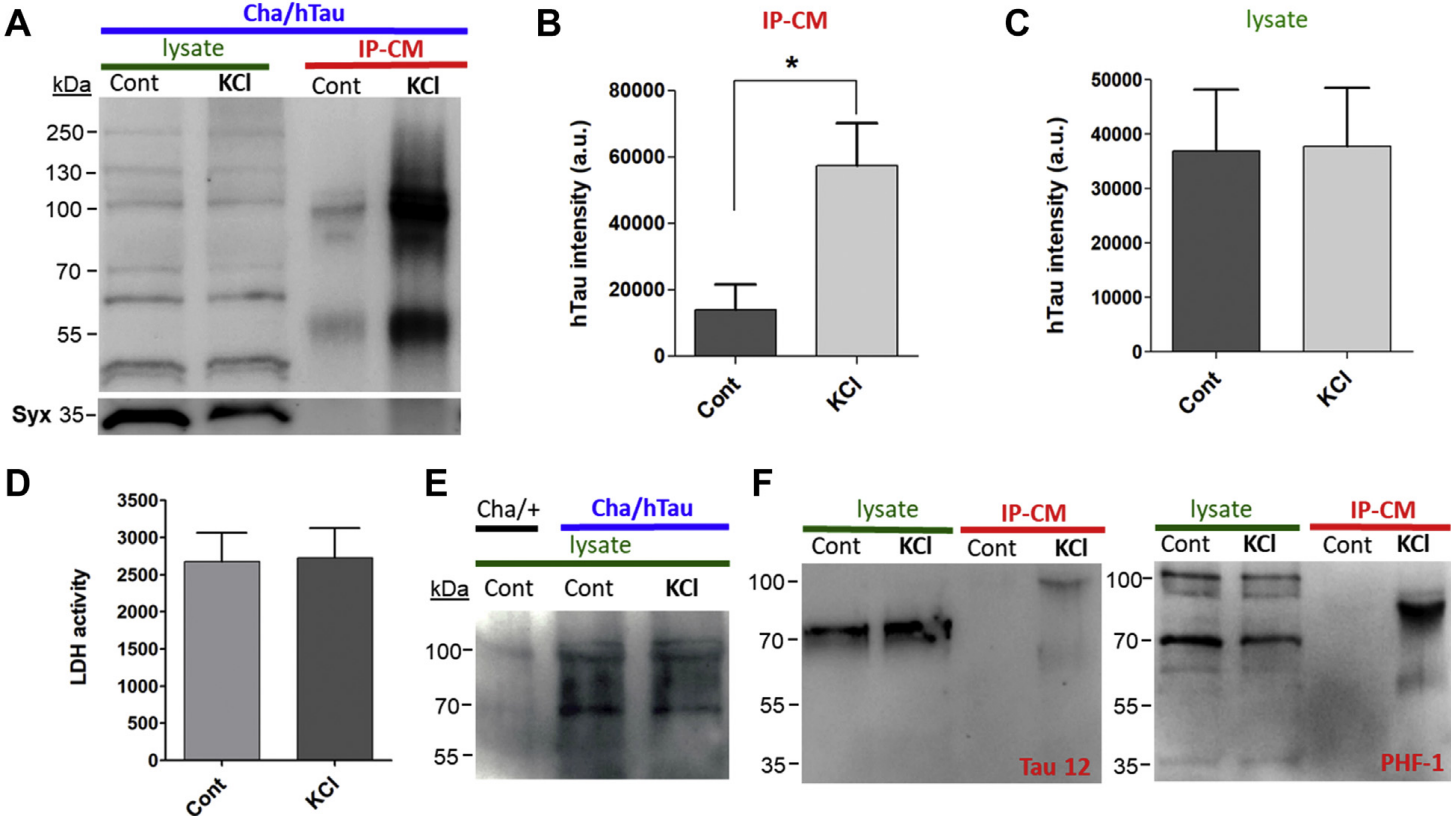
**KCl-induced neuronal depolarization increases release of human tau (hTau).***A*, representative Western blot (WB) image showing intracellular hTau in lysate and hTau immunopurified from conditioned media (IP-CM). hTau was released to the culture media after 50 mM KCl treatment for 1 h 9 days *in vitro* (DIV) culture (ChaGFP-Gal4 × hTau^2N4R^) was used. hTau bands quantified in CM and lysate were between 55 and 100 kDa. *B*, quantification of HT7 intensity in IP-CM from control and KCl-treated sample groups (n = 3). *C*, quantification of HT7 intensity in cell lysate (n = 3). *D*, lactate dehydrogenase (LDH) activity in the CM was unchanged by 50 mM KCl treatment (n = 6). *E*, representative WB shows HT7 antibody does not interact with fly endogenous tau. *F*, representative WB shows that a small fraction of tau released by activity is detected by Tau 12 antibody, whereas released tau is strongly stained by PHF-1 antibody. ∗*p* < 0.05, Student's *t* test. PHF, paired helical filament.

Using the aforementioned primary cultures expressing hTau^2N4R^ (ChaGFP-Gal4 × UAS-hTau^2N4R^), we examined if tau release could be detected in the media of resting neurons (constitutive release) and if release could be increased by neuronal activity (*i.e.*, KCl-induced depolarization). Culture medium was harvested after 50 mM KCl treatments for 1 h. Immunopurified conditioned media (IP-CM) showed significantly higher hTau levels compared with IP-CM from resting neurons incubated for 1 h with physiological concentrations of KCl (5.6 mM) by Western blot analysis (Fig. 2, *A* and *B*).

Lysate tau bands are observed in the range of 40 to 250 kDa. In contrast, released tau proteins in the IP-CM were observed in the 55 to 100 kDa range, regardless of which tau antibody was used (*e.g.*, HT7 or phospho-specific tau antibodies). Therefore, for comparison purposes, tau signals between 55 and 100 kDa were densitometrically measured for both lysate and IP-CM tau. The causes for the differences in molecular weight were not determined in this study although it might be due to post-translational modification of tau such as truncation (see later).

The band at 68 kDa is expected for monomeric full-length tau (2N4R). The presence of higher molecular-weight tau bands suggests that both hTau monomers and dimers are released during KCl-induced depolarization. Western blots of cell lysates showed several tau bands in the range of 40 to 250 kDa as well. Higher molecular-weight tau bands in lysates could be tau dimers and oligomers (*e.g.*, 100, 130, and 250 kDa in Fig. 2*A*), supporting the existence of intracellular oligomers/aggregates in neurons, as suggested by immunofluorescence (Fig. 1, *B* and *C*).

Expression levels of hTau were judged similar when comparing Western blots of lysates from control and KCl-treated cultures normalized to syntaxin in the same blot (Fig. 2, *A* and *C*), suggesting that the increased levels of hTau in IP-CM were not because of differences in neuronal expression levels of hTau. We next sought to determine whether hTau found in IP-CM was released by neurons as a result of cell damage resulting from KCl-induced depolarization. Lactate dehydrogenase (LDH) activity in the CM was measured to evaluate the contribution of cell damage to increased tau release. No significant differences were observed in LDH activity assayed in CM when comparing 50 mM KCl with physiological KCl (Fig. 2*D*). The results demonstrate that the increase in hTau release during KCl-induced depolarization is not because of nonspecific cell damage. We also confirmed that the anti-HT7 antibody shows high selectivity to hTau *versus* endogenous fly tau (Fig. 2*E*). ChaGFP-Gal4 was crossed with wildtype flies, and thus, no hTau expression is expected.

To further characterize released tau, we used two additional antibodies, Tau12 and PHF-1 antibodies, which recognize N and C termini of tau, respectively. PHF-1 also recognizes pSites (396/404) in C-terminal domain of tau. As shown in Figure 2*F*, much of lysate and released tau appears to be N-terminal truncated because Tau12 antibody failed to recognize tau protein bands except one approximately 70 kDa (presumably full-length tau). In contrast, intracellular and released tau was strongly detected by PHF-1.

### Phosphorylation state influences depolarization-dependent hTau release

Next, we investigated the role of tau phosphorylation in depolarization-induced hTau release. Two fly lines were used that expressed either a pseudophosphorylated hTau^E14^ or a phosphoresistant hTau^S11A^. A transgene hTau^E14^ carries mutations (serine/threonine to glutamate) in 14 disease-associated pSites, which mimic a hyperphosphorylation state and play a role in tau toxicity (25). In contrast, hTau^S11A^ has 11 of 14 mutations (serine/threonine to alanine) in GSK3β homolog *Shaggy* pSites, including phospho-specific AT8, AT100, AT180, and PHF1 epitopes (26).

**Figure 3:**
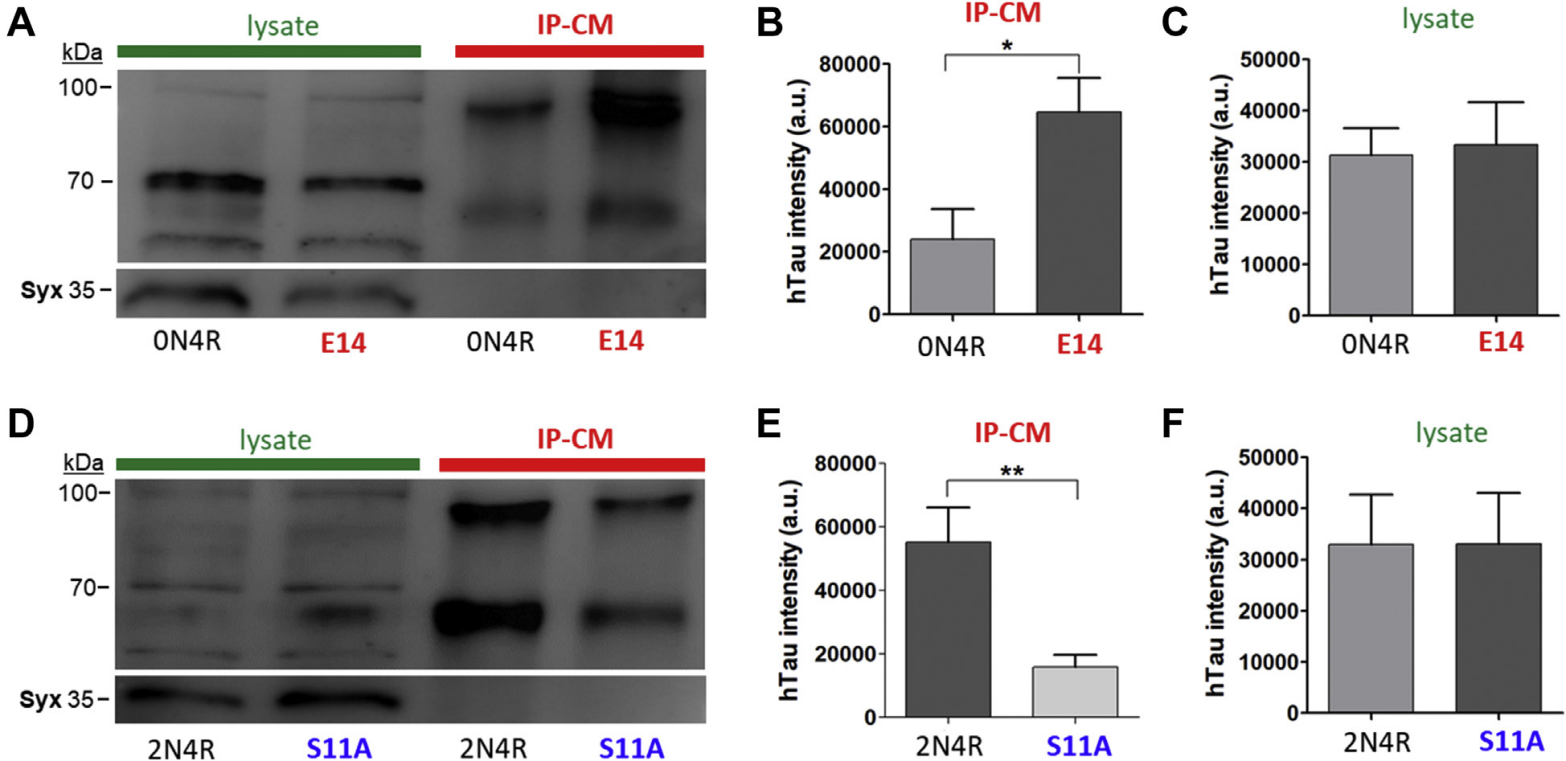
**Phosphorylation influences depolarization-dependent human tau (hTau) release.***A*, representative Western blot (WB) image of hTau in immunopurified conditioned media (IP-CM) from 9 DIV neuronal culture of hTau^0N4R^ (ChaGFP-Gal4 × UAS-hTau^0N4R^) and E14 (ChaGFP-Gal4 × UAS-hTau^E14^) after 50 mM KCl treatment for 1 h. *B*, quantification of released hTau^0N4R^ and E14 in the IP-CM. *C*, quantification of hTau^0N4R^ and E14 in cell lysates. n = 4 for (*B*) and (*C*). *D*, representative WB of hTau in IP-CM from 9 DIV culture of hTau^2N4R^ (ChaGFP-Gal4 × hTau^2N4R^) and S11A (ChaGFP-Gal4 × UAS-hTau^S11A^) after 50 mM KCl for 1-h treatment. *E*, quantification of released hTau^2N4R^ and S11A in IP-CM. *F*, quantification of hTau^2N4R^ and S11A in cell lysates. Anti-HT7 antibody was used to probe intracellular and released hTau. n = 4 for (*E*) and (*F*). Western bands of hTau between 55 and 100 kDa were quantified in cell lysate and CM. ∗∗*p* < 0.01 and ∗*p* < 0.05. Student's *t* test.

These genetic tools allowed a direct comparison of the effect of phosphorylation or dephosphorylation in the proline-rich domain (PRD) and C-terminal domain of hTau upon its depolarization-induced release. ChaGFP-Gal4 was used to drive the expression of UAS-hTau^E14^, UAS-hTau^S11A^, wildtype hTau 0N4R (UAS-hTau^0N4R^) and hTau 2N4R isoforms (UAS-hTau^2N4R^), respectively. E14 tau was mutated from 0N4R wildtype tau isoform, whereas S11A was from 2N4R. Therefore, we used 0N4R and 2N4R as controls for E14 and S11A, respectively. When hTau^E14^ was expressed, depolarization-dependent release was significantly increased when compared with neurons expressing hTau^0N4R^ (Fig. 3, *A* and *B*). To test the effect of the lack of phosphorylation in PRD and C-terminal domain, neurons expressing hTau^S11A^ were studied and showed reduced depolarization-induced release when compared with neurons expressing hTau^2N4R^ (Fig. 3, *D* and *E*). The results suggest that phosphorylation in these domains of the hTau enhances release, whereas lack of phosphorylation decreases release. This apparent phosphorylation-dependent hTau release was not associated with changes in intracellular hTau expression levels as determined by Western blots of cell lysates (Fig. 3, *C* and *F*). This study raised an intriguing question—is endogenously phosphorylated hTau released, and if so, is phosphorylated hTau the preferred substrate for release (see later)?

### hTau release was increased by optogenetic stimulation

As our results suggested that hTau release is regulated by neuronal depolarization in *Drosophila* neurons, we were prompted to confirm the depolarization-induced hTau release *via* an optogenetic approach, which provides a highly controlled and precise method of inducing cellular depolarization. Optogenetic stimulation is physiologically relevant and allows stimulation to be targeted to specific neurons.

**Figure 4:**
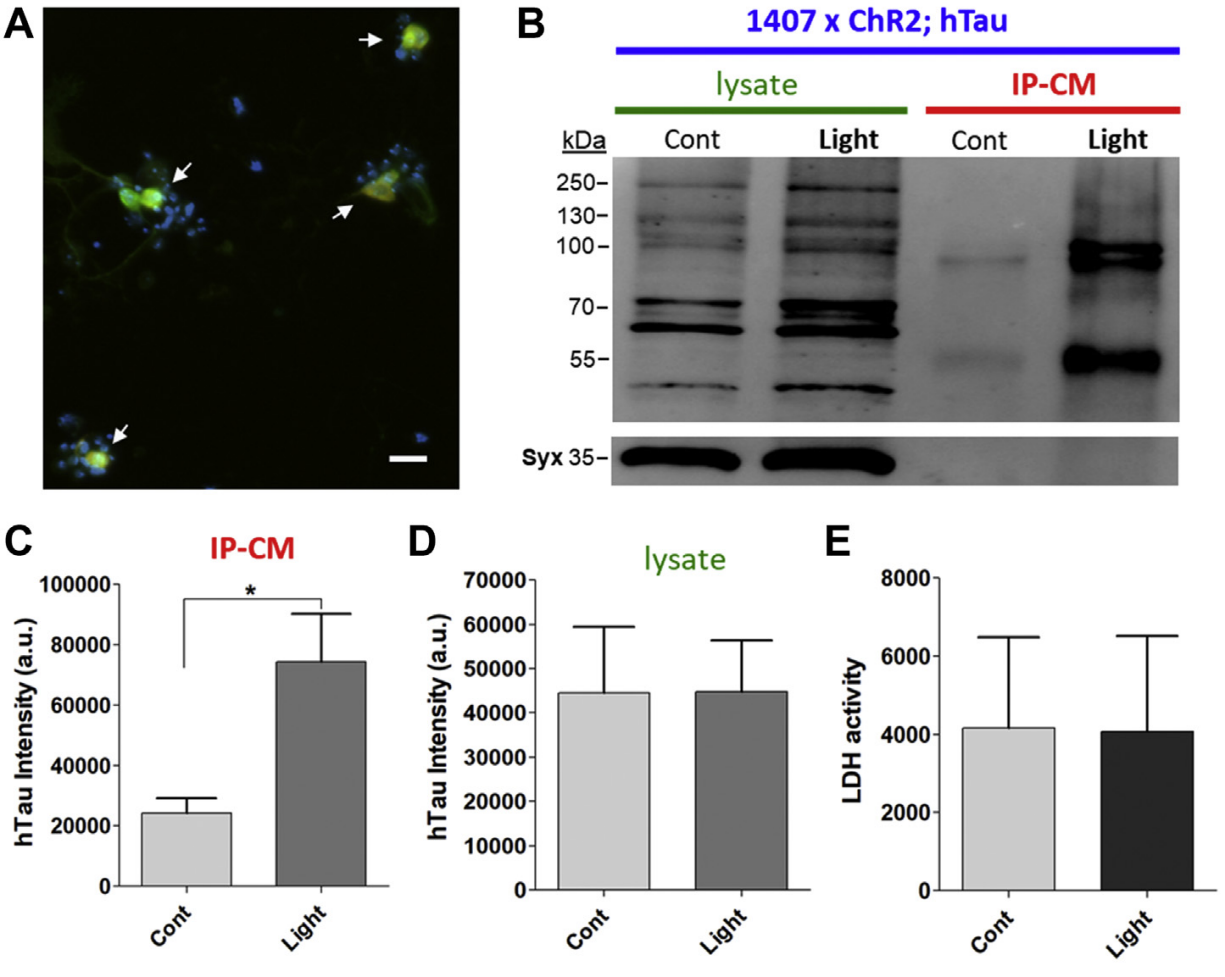
**Activity-dependent human tau (hTau) release by optogenetic stimulation.** Neuronal culture was prepared from a cross line of 1407-Gal4 × UAS-ChR2_mCherry; hTau^2N4R^. All cultures were incubated with 2.5 μM all-*trans* retinal (ATR) for 2 days before stimulation by blue light (BL; 470 nm) for 30 min. *A*, primary cultured neurons (3 DIV) expressing ChR2_mCherry and hTau^2N4R^, detected by anti-mCherry (*red*) and HT7 antibodies (*green*). Neurons expressing both mCherry and hTau are indicated by *arrows*. The scale bar represents 10 μm. *B*, representative Western blot image showing hTau from lysate and IP-CM. High molecular tau bands (≥130 kDa) were observed as seen for Figure 2*A*. *C*, quantification of hTau bands from IP-CM (n = 5). ∗*p* < 0.05, Student's *t* test. *D*, quantification of HT7 intensity in cell lysate (n = 3). hTau bands quantified were between 55 and 100 kDa. *E*, LDH activity in the conditioned media was not changed by BL exposure (n = 3). IP-CM, immunopurified conditioned media; LDH, lactate dehydrogenase.

In this study, we used a pan-neuronal driver 1407-Gal4 in order to observe tau release from a general population of neurons. Cultured neurons expressing hTau and channelrhodopsin 2 (ChR2) were obtained by crossing 1407-Gal4 and UAS-ChR2_mCherry; UAS-hTau^2N4R^ lines. ChR2 is gated by blue light (BL; 470 nm), thus neurons expressing ChR2 are expected to be depolarized when exposed to BL. First, we confirmed coexpression of ChR2_mCherry and hTau^2N4R^ in the same neurons cultured from 1407-Gal4 × UAS-ChR2_mCherry; hTau^2N4R^ (Fig. 4*A*). Since fly neurons do not make all-*trans* retinal (ATR), a cofactor of ChR2, primary neuronal cultures were treated with 2.5 μM ATR at 7 DIV for 2 days. Subsequent exposure to BL for 30 min resulted in increased hTau release when compared with no BL control (Fig. 4, *B* and *C*). In contrast, BL exposure for 30 min resulted in no change in LDH release (Fig. 4*E*) when compared with no BL, demonstrating that BL exposure does not damage neurons. These results strongly confirm that neuronal depolarization increases hTau release.

### Is released hTau phosphorylated?

**Figure 5:**
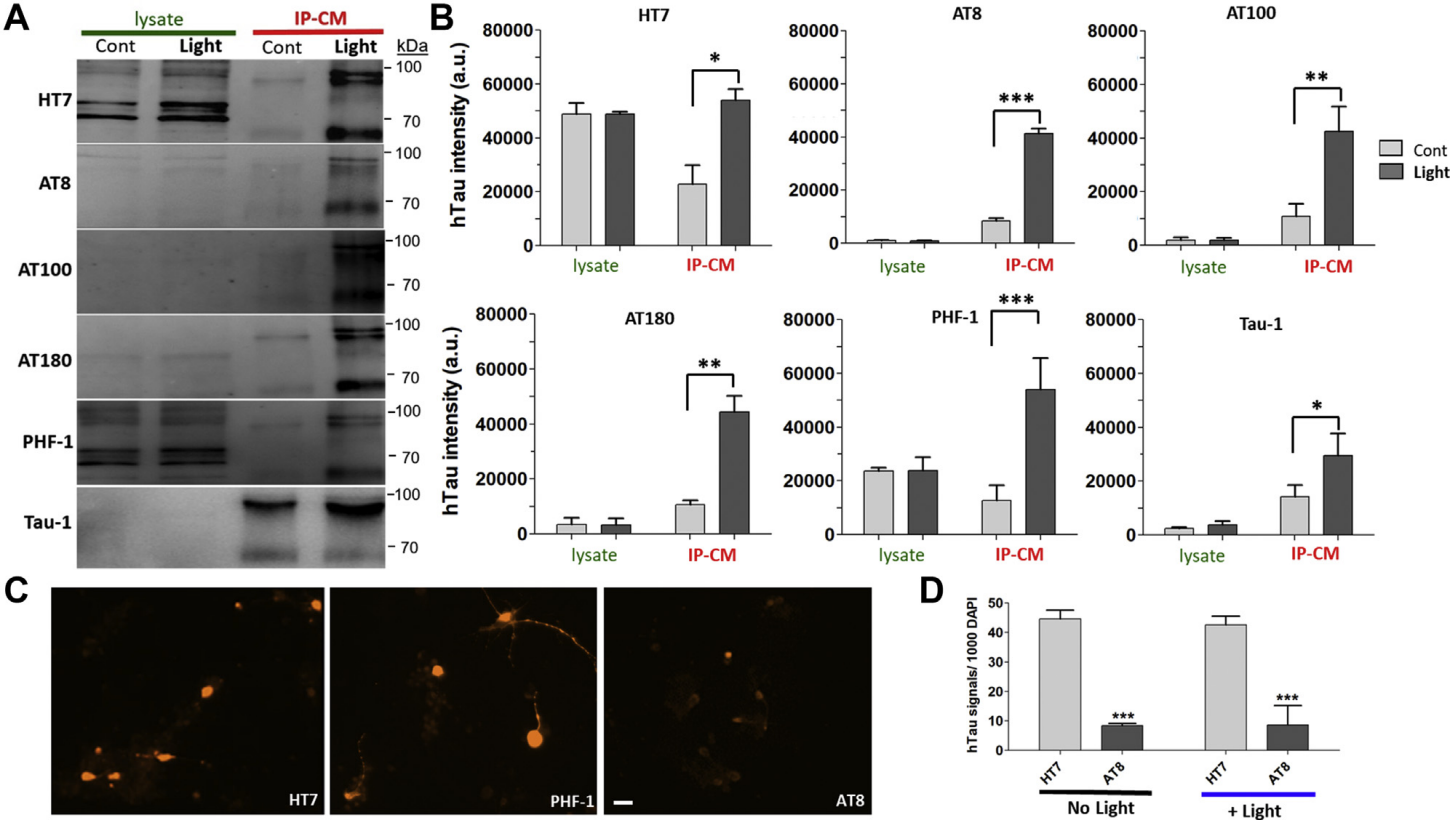
**Phosphorylated tau is preferentially released.***A*, representative Western blot (WB) image showing human tau (hTau) bands from lysate and IP-CM. Membranes were probed with total hTau (HT7) and phosphorylation site-specific hTau antibodies, AT8, AT100, AT180, and PHF-1. Tau-1 antibody was also used to detect unphosphorylated site(s) of hTau in lysate and IP-CM. The HT7 panel (*top*) is reproduced from Figure 4*B* for the purpose of better visual reference with the other WB images used in this figure. *B*, WB quantification of lysate and CM bands from 1407-Gal4 × UAS-ChR2_mCherry; hTau^2N4R^ neuronal cultures (9 DIV). Quantification of hTau bands between 55 and 100 kDa (cont *versus* light). hTau signals in lysate and CM to each antibody were quantified relative to syntaxin (syx) in cell lysate (refer to Fig. 4*B*). *C*, neuronal cultures (7–9 DIV) were stained with HT7, AT8, and PHF-1 antibodies. The scale bar represents 15 μm. *D*, quantification of HT7 and AT8-positive neurons in cultures with or without light stimulation. Cultures were stimulated by exposing to blue light (470 nm) for 30 min. Data from three to five separate cultures. ∗*p* < 0.05, ∗∗*p* < 0.01, ∗∗∗*p* < 0.001, Student's *t* test. IP-CM, immunopurified conditioned media; PHF-1, paired helical filament 1.

Phosphorylation of tau plays an important role in activity-dependent tau release and suggested that endogenously phosphorylated hTau is released during depolarization (Fig. 3). Thus, we sought to determine the phosphorylation state of released hTau using pathologically relevant pSite-specific antibodies (Fig. 5): S202/T205 (AT8), T212/S214 (AT100), T231 (AT180) in the PRD, and S396/404 (PHF1) in the C-terminal domain. Since a few studies (14, 27) showed that tau released from rat primary neuronal cultures during depolarization is dephosphorylated at S195/198/199/202, we also examined released tau with Tau-1 antibody. HT7 antibody, which shows no preference for either phosphorylated or dephosphorylated hTau, was used to measure the amount of total tau released.

We used 8 to 9 DIV culture from 1407-Gal4 × UAS-ChR2_mCherry; hTau^2N4R^. After 30 min of stimulation by BL, CM from both control and BL-exposed neurons were harvested and then purified by immunopurification (IP-CM) before Western blot analysis. The data confirm that much greater amounts of hTau were immunopurified from CM obtained from neurons exposed to BL compared with no BL control regardless of the antibody used (Fig. 5, *A* and *B*). Interestingly, hTau protein phosphorylated at these same sites within PRD were barely detectable in cell lysates (Fig. 5, *A* and *B*). This suggests that intracellular hTau overexpressed in these neuronal cultures was largely lacking phosphorylation at the sites probed except PHF-1. To further confirm this finding, intracellular tau in cultured neurons was visualized using a phospho-specific antibody AT8 and immunofluorescence (Fig. 5*C*). When compared with HT7-positive neurons, there were significantly fewer neurons that reacted strongly with the AT8 antibody (Fig. 5*D*). In contrast, the PHF-1 antibody recognized tau in cultured neurons as well as tau on Western blots obtained from cell lysates (Fig. 5, *A*–*C*). Interestingly, released tau appeared to be unphosphorylated at pSites 195, 198, 199, and/or 202. Tau-1 intensity in IP-CM was also increased with BL stimulation (Fig. 5, *A* and *B*).

Our data (Fig. 5, *A* and *B*) show that the total intracellular hTau pool expressed in fly neurons is similar in control and light-treated groups. However, fold increases of phosphorylated hTau signals in IP-CM after light stimulation were greater than that of total tau HT7 or unphosphorylated Tau-1 signals (Table 2). This apparent enrichment of phosphorylated hTau in IP-CM obtained from the neuronal cultures exposed to BL strongly suggests that hTau phosphorylated at these sites is released in large quantities (Fig. 5, *A* and *C*). We conclude that phosphorylated tau is preferentially released to the media with neuronal activity.

**Table 2:** Fold changes of Western blot hTau signals derived from either lysate or IP-CM

Tau epitope	Lysate	IP-CM
HT7	1.0	2.48
AT8	0.95	4.89
AT100	1.05	3.91
AT180	0.96	4.18
PHF-1	1.0	4.23
Tau-1	1.52	2.06

Fold changes were calculated as the ratio of hTau measured after light stimulation *versus* no light stimulation for each antibody listed. Data derived from results are seen in Figure 5*B*.

## Discussion

*Drosophila* is a well-established animal model used to study human neurodegenerative diseases (28, 29, 30, 31) with numerous and readily available transgenic lines and sophisticated genetic tools such as Gal4/UAS system (23, 32). Using *Drosophila* neurons in primary culture, we show that hTau release is greatly increased by depolarization, induced by either high KCl treatment or optogenetic stimulation. We have verified that *Drosophila* neuronal culture combined with optogenetics offers a highly tractable model of hTau release and can be an extremely valuable model to study mechanisms underlying activity-dependent hTau release.

Tau phosphorylation has profound impacts on its physiological and pathological functions (5, 17, 33). Under normal conditions, phosphorylated tau can be released (14, 34, 35) although the physiological function of released tau needs further study. Hyperphosphorylated tau is released under pathological conditions as well, and its hyperphosphorylation (*e.g.*, E12 tau; 12) is known to play a key role in tau propagation and pathology. Our results showed that phosphorylation of hTau appears to modulate its activity-dependent release as the transgenic line carrying phospho-resistant hTau showed reduced activity-dependent release compared with wildtype hTau, whereas the pseudophosphorylated hTau line showed a significant enhancement of activity-dependent release. This is in agreement with a study performed with HeLa cells in which they reported mimicking phosphorylation at 12 sites (E12) known to be involved in AD-enhanced tau release (21). Recently, Katsinelos *et al.* (36) showed that abnormally phosphorylated tau is preferentially released from CHO and SH-SY5Y cells.

Pooler *et al.* (14) showed that endogenous tau release from mouse primary cortical neurons is increased when neurons are depolarized; this finding is consistent with our findings. However, the phosphorylation state of released tau is still controversial (5, 14, 17). Pooler *et al.* (14) concluded that released endogenous tau was unphosphorylated based on Western blot analysis with antibodies that preferentially recognize unphosphorylated tau (Tau-1) or phosphorylated tau (PHF-1). Mohamed *et al.* (27) have shown that tau release from resting mouse neurons, which was not activity dependent, is dephosphorylated. Recently, Croft *et al.* (37) showed that resting tau release from organotypic brain slice cultures from the 3xTg-AD mouse model was dephosphorylated as well. Released tau can be dephosphorylated at pTau199 site, which is consistent with our results showing strong Tau-1 Western signals in the IP-CM (Fig. 5, *A* and *B*).

However, other studies (22, 38, 39) showed that released tau is phosphorylated. Karch *et al.* (40) showed that extracellular tau released from human neuroblastoma cells (SH-SY5Y) is phosphorylated at several epitopes: T181 and S396. Saman *et al.* (38) also showed that M1C cells overexpressing tau^0N4R^ secrete phosphorylated tau selectively. Most recently, Wadhwani *et al.* (41) reported that endogenous tau is released from neurons derived from human-induced pluripotent stem cells, and pSites of released tau include T181, T231, and S396, but interestingly, S199 was not detected, which is consistent with the previous findings (14, 27, 37). Our results also showed that hTau released from *Drosophila* neurons was phosphorylated at several sites (AT8, AT100, and AT180) in PRD, demonstrating that released hTau is phosphorylated under these conditions. In addition, tau in CSF and blood is phosphorylated in patients with AD (42, 43). Interestingly, the presence of these epitopes in CSF (*e.g.*, p181) distinguishes between AD and non-AD pathology, and thus, phosphorylated tau released from neurons into CSF can be a predictor of progressive cognitive impairment and presymptomatic patients (42, 44, 45).

Released tau is unphosphorylated at Tau-1 sites, but more pSites are phosphorylated (Fig. 5, *A* and *B* and Table 2). Discrepancies between reports on phosphorylated- or unphosphorylated-released tau can be related to model systems used (*e.g.*, rodent *versus* human cells), endogenous *versus* overexpressed tau, releasing methods (*e.g.*, membrane free or vesicle bound), physiological *versus* pathological tau, and others. Additional studies are required to resolve the discrepancies. However, it should be noted that most studies reporting unphosphorylated tau used a small number of phospho-specific or dephospho-specific antibodies (*e.g.*, Tau-1) and thus provide an incomplete understanding of all the potential phosphorylated species of released tau regardless of its constitutive or activity-dependent release. In addition, it is interesting that taus released from all human cells including neural cell lines are phosphorylated at multiple sites (38, 39, 40, 41). Díaz-Hernandez *et al.* (46) also showed that phosphorylated tau is released from SH-SY5Y neuroblastoma although released tau is dephosphorylated by tissue-nonspecific alkaline phosphatase after release. hTau released from *Drosophila*-cultured neurons is phosphorylated at multiple pSites and unphosphorylated at Tau-1 sites (Fig. 5). Therefore, it is important to systemically exam pSites in released tau, which will help us to better understand the role of phosphorylation in tau release and also phosphorylation status of released tau in healthy and diseased conditions.

In our study, phosphorylation of intracellular tau at pSites recognized by AT8, AT100, and AT180 antibodies was not detectable by Western blot (Fig. 5). We believe this finding is not because of endogenous phosphatases in cell lysates due to the following reasons. First, we examined another phospho-specific antibody, PHF-1 (396/404). Without including phosphatase inhibitors (PIs), PHF-1 detected high levels of intracellular tau (Fig. 5, *A* and *C*). Second, we also stained intracellular tau in cultured neurons using AT8 antibody (Fig. 5, *C* and *D*). Compared with HT7-positive neurons, much less AT8-positive neurons were observed. Third, we examined levels of phosphorylated tau at S202/T205 with AT8 antibody (Fig. S1) in the presence and absence of PIs. Although AT8 signal in lysates was slightly higher in the presence of PIs, this increase was not significant enough to influence our conclusion that phosphorylated tau is preferentially released from *Drosophila* neurons (Fig. 5).

Our study is the first to show that neuronal stimulation increases release of hTau phosphorylated at AT8, AT100, AT180, and PHF-1 sites. Although we have discovered a putative relationship between phosphorylation of hTau and its activity-dependent release, little is known about the impacts of specific protein kinases on activity-dependent tau release. Specific tau kinases that phosphorylate distinct tau pSites in PRD and C-terminal domain would differentially modulate activity-dependent hTau release. Therefore, a logical outgrowth of our findings is to identify the protein kinases (*e.g.*, GSK-3β, CDK5) involved in our observations, as this will provide novel insights into the pathophysiological significance of this phenomenon.

Our study is likely to have high impact on ongoing efforts to understand molecular mechanisms of cellular release and cell-to-cell propagation of tau, and how neuronal hyperexcitability and increased synaptic activity are involved in the progression of AD. Recent studies showed using a tau tracer (*i.e.*, AV1451-PET), tau spreading is closely related to the progression of preclinical AD (47). Therefore, our approach will likely contribute to efforts developing novel therapeutic strategies. Furthermore, high- and medium-throughput screens are easily achieved in *Drosophila* neuronal culture to identify novel molecules able to modify activity-dependent tau release. Thus, small-molecule drug candidates can be rationally designed and targeted to critical molecular steps controlling tau release and thus reducing tauopathies.

It is known that Aβ causes hyperexcitability in the brain (48). We showed that neural stimulation enhances tau release and thus likely spreading of tauopathies. It will be of interest to study the relationship between Aβ and tau release in the progression of neurodegeneration. In addition, recent studies showed that Aβ plaques and tau tangles can alter functional connectivity of networks (*e.g.*, default-mode network) involving cognition (47, 49). Therefore, understanding mechanisms underlying activity-dependent tau release and propagation can be a key step forward to unraveling how functional connectivity networks in the brain are altered and degenerate.

Our current study primarily focuses on the role of tau phosphorylation and pSites in activity-dependent release. However, several important questions must be addressed in future studies. First is to understand the role of these individual phosphorylated sites in the mechanism of tau release and disease propagation. Because of the variety of combinations of pSites that could be tested, it is preferable to identify and test pSites on the basis of a link between pSites (*e.g.*, S11A or E14) and AD pathology. Since released tau is phosphorylated at multiple sites (epitopes of AT8, AT100, AT180, and PHF1) (Fig. 5), it is possible that phosphorylation of multiple pSites, rather than single pSites, is important for increasing activity-dependent tau release. Since some of tau kinases are known to phosphorylate more than one pSite (2, 17, 33), studies combining protein kinase action with genetic modification of individual tau pSites will yield a better understanding of the role of individual pSites in activity-dependent tau release. Second, uptake of extracellular tau is poorly understood while being a presumed critical step in transneuronal propagation. Recently, Rauch *et al.* (50) showed that low-density lipoprotein receptor–related protein 1 controls tau endocytosis. Therefore, *Drosophila* primary neuronal culture (Fig. 1*D*) can be a useful system to identify additional genes important for tau uptake because of its availability of sophisticated genetic models. Third, phosphorylated tau could be released physiologically from healthy neurons as an activity-dependent process (13, 14). Therefore, it should be determined if the tau release observed in *Drosophila* neuronal culture is physiological or pathological. Physiological neuronal activities can induce tau release under healthy conditions, but when neurons are hyperstimulated, released tau could play a role in excitotoxicity and neurodegeneration. It is possible that different forms of tau (monomeric *versus* oligomeric) may be released in normal as well as pathological conditions. It is still unknown which forms of tau mediate its cell-to-cell propagation (4). Fourth, hTau (0N4R or 2N4R isoform) in this study is heterologously expressed in *Drosophila*-cultured neurons. Although studies showed that endogenous tau is also released from neurons (14, 51), we cannot yet conclude that endogenously expressed hTau (mixture of six isoforms) can be released in activity- and phosphorylation-dependent manner. Finally, whether mechanisms of tau release and propagation in *Drosophila* primary culture are directly applicable to human models needs verification. Recently, human neural progenitor cell lines became a powerful *in vitro* model to study Alzheimer's pathology (51, 52). Therefore, our *Drosophila* cultures can be used in conjunction with human neural progenitor cell lines to further confirm findings in *Drosophila* culture and its translational value.

## Experimental procedures

### Fly stocks

Flies are kept in a standard cornmeal/agar medium with 0.4% propionic acid at 25 °C in a 12 h light/dark cycle. The following are the fly strains used in this study: Cha-Gal4, 1407-Gal4, UAS-hTau^2N4R^, UAS-hTau^S11A^, UAS-GFP, UAS-mCherry_ChR2 (Bloomington *Drosophila* stock center), UAS-hTau^0N4R^, and UAS-hTau^E14^ (kind gifts from Dr M. Feany, Harvard Medical School).

### Drosophila primary neuronal cultures

Drosophila primary neuronal cultures were prepared and incubated in 5% CO_2_ at 25 °C as previously described (53, 54). Half of the culture medium was replaced to keep the culture healthy every 4 to 5 days. Cells in *Drosophila* primary cultures are predominantly neurons (55).

### Immunofluorescence assay

Immunofluorescence assay was performed as previously described (53, 54). For hTau visualization, mouse antitotal hTau antibody (HT7; 1:1000) was used. To visualize phosphorylated tau, mouse antiphosphorylated hTau antibodies (AT8, AT100, AT180 [Thermo Fisher Scientific], and Tau-1 [Millipore Sigma]) were used. PHF-1 is a kind gift of Dr Peter Davies at the Feinstein Institute for Medical Research in Manhasset, NY. Tau 12 (Millipore Sigma) was used to detect the N-terminal end of tau. Primary antibodies were incubated overnight at 4 °C and detected with secondary antibodies for 1 h on ice (FITC or tetramethylrhodamine labeled; Invitrogen). Coverslips were mounted on glass slides and imaged using epifluorescence (Olympus 1X71). Images were captured by spot CCD digital camera (Diagnostic Instruments).

To determine the number of tau-positive neurons (*i.e.*, HT7 or AT8 antibody) in our cultures, we counted the number of cells stained by an antibody per 1000 cells in each treatment group. Cells are counted using a nuclear dye 4′,6-diamidino-2-phenylindole (Molecular Probes) as previously described (53).

### KCl stimulation

At 7 to 9 DIV, culture coverslips were transferred into 24-well plates with either physiological KCl solution (in millimolar): 154 NaCl, 5.6 KCl, 2.3 CaCl, 1 MgCl_2_, 5 Hepes, 10 glucose, pH 7.4 or 50 mM KCl solution (in millimolar): 110 NaCl, 50 KCl, 2.3 CaCl, 1 MgCl_2_, 5 Hepes, 10 glucose, and pH 7.4. After 1 h, the media were carefully transferred into separate prechilled eppendorf tubes on ice. The CM was pooled from four coverslips and centrifuged for 10 min at 10,000 rpm to remove cell debris. The supernatant fluid was collected and immunopurified (IP-CM, see later) in preparation for Western blot analysis. The same coverslips were then used for lysate preparation (total cellular protein).

### IP

Pierce crosslink IP kit (Thermo Fisher Scientific) was used to immunopurify hTau protein from the CM. Thus, in this study, we are measuring changes in free and not membrane-bound hTau. The binding of HT7 to protein A/G plus agarose was performed according to the Pierce crosslink IP kit protocol. CM was collected and incubated with HT7 antibody–crosslinked beads overnight at 4 °C. After discarding the column flow-through, the beads were washed twice with IP lysis/wash buffer and once with conditioning buffer (neutral pH). The immunocomplexed protein was eluted with 10 μl elution buffer to a new eppendorf tube with centrifugation. Then, beads were incubated in an additional 50-μl elution buffer for 5 min at room temperature. Finally, the column was centrifuged, and the second flow-through was pooled with the first IP-CM elution.

### Western blot analysis and quantification

Lysates and IP-CM were loaded on SDS-polyacrylamide gels for electrophoresis. PageRuler Plus prestained protein ladder (Thermo Fisher Scientific) was used to estimate protein molecular weight. Using analyze gel function in the ImageJ program (National Institutes of Health), Western blot band intensities were densitometrically quantified and compared between experimental groups (lysates and IP-CM of control *versus* experimental groups).

Each Western blot analysis of tau release represents samples pooled from four coverslips obtained from a single neuronal culture (Figure 2, Figure 3, Figure 4, Figure 5; lysate [cont], lysate [KCl or light], IP-CM [cont], and IP-CM [KCl or light]). Lysate densitometric intensities were normalized to syntaxin, a pan-neuronal expressed protein in *Drosophila* (56). Variations in culture density, although purposely kept small, could contribute to the observed differences in IP-CM hTau levels detected in Western blots. To remove this potential error, we adjusted the measured intensities of IP-CM Western blot lanes by total lysate protein of the source coverslip using the measured syntaxin level for that lane.

For Western blot analysis with phospho-specific tau antibodies (*e.g.*, AT8), endogenous phosphatase activities, both intracellular and/or extracellular could affect Western blot results. We compared lysate phosphorylated tau levels in the absence and presence of PIs (Halt protease and PI cocktail; Thermo Fisher Scientific). Very low levels of phosphorylated hTau in lysates were seen in the absence or the presence of PIs (Fig. S1). Phosphorylated tau was not increased in lysates with the inclusion of PIs. Tau protein levels were similar in all lanes used for the analysis (HT7 antibody). Thus, PIs were not included in the analysis reported for Figure 2, Figure 3, Figure 4, Figure 5. All experiments were carefully processed in the same way with the same time course and temperature, reducing the likelihood that observed effects were due to harvesting/processing artifacts.

### Optogenetics

Culture coverslips were incubated with 2.5 μM ATR for 2 days and then transferred to 24-well plates. BL (470 nm) was shed on the coverslips for 30 min in the CO_2_ incubator, whereas the control coverslips were kept in a box isolated from BL. After BL, the media were collected, pooled from four coverslips, and carefully transferred to a prechilled tube on ice. Pooled CM was centrifuged for 10 min at 10,000 rpm. The supernatant was used for IP. Coverslips were then treated for lysate preparation (total cellular protein).

### Lysate preparation

Neuronal cultures were homogenized in 100 μl radioimmunoprecipitation assay buffer (Thermo Fisher Scientific) supplemented with protease inhibitor cocktail (Roche) on ice. The lysate was then centrifuged for 10 min at 10,000 rpm at 4 °C. The supernatant was transferred to a fresh prechilled tube and kept frozen at −70 °C until used for Western blotting.

### Statistical analysis

Bar graphs depict the mean ± SEM of biological replicates (n = the number of replicates), each representing separate neuronal cultures prepared on different days (see the legends to the figures). Each assay or sample used for Western blot analysis is derived from pooling several coverslips. Statistical analysis was performed using GraphPad Prism software (GraphPad Software, Inc). Means were compared using an unpaired two-tailed Student's *t* test. *p* values ≤0.05 were considered significant.

## Data availability

All data are contained within the article.

## Supporting information

This article contains supporting information.

## Conflict of interest

The authors declare that they have no conflicts of interest with the contents of this article.
