# Supplementary material for: Activity-dependent release of phosphorylated human tau from Drosophila neurons in primary culture
Source: J Biol Chem. 2021 Aug 30;297(4):101108. doi: 10.1016/j.jbc.2021.101108 (PMC8455371; doi:10.1016/j.jbc.2021.101108)
Supplement: Figure S1 [file mmc1.docx]

Supporting Information


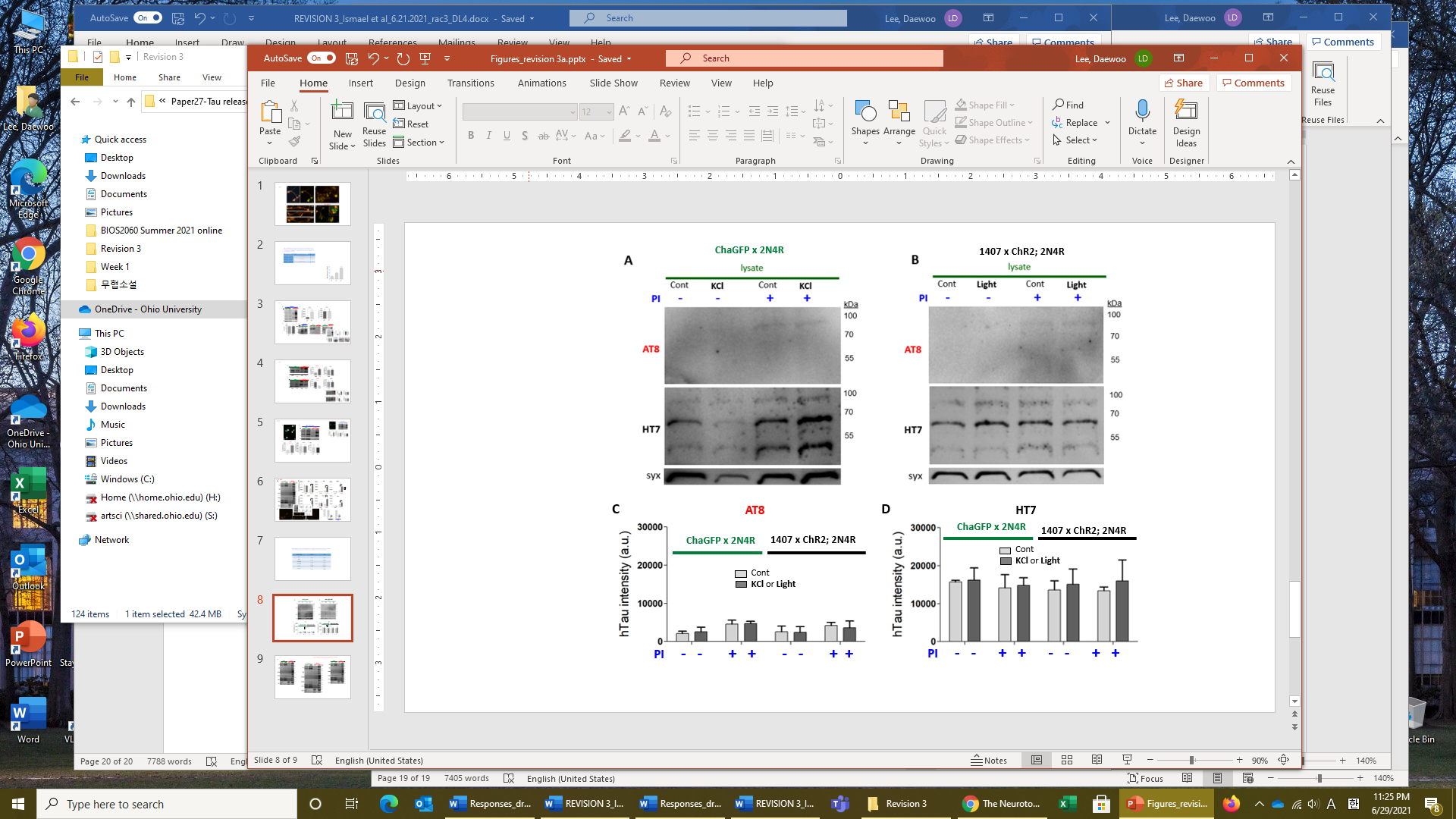


**Figure S1. Western blot analysis of phosphorylated tau in lysates is unaffected by the absence and presence of phosphatase inhibitors (PI). (A & B)** Western blot images of AT8 signal from lysates (Cont. versus KCl- or light-stimulated) of ChaGFP x 2N4R (A) and 1407-Gal4 x UAS-ChR2_mCherry; 2N4R cultures (B) in the absence and presence of phosphatase inhibitors (PI). **(C)** AT8 signal quantification of lysate bands from ChaGFP x 2N4R and 1407-Gal4 x UAS-ChR2_mCherry; hTau^2N4R^ neuronal cultures. In the presence of PI, AT8 signals were slightly increased but this increase was not statistically significant. **(D)** HT7 signal quantification of lysate bands from ChaGFP x 2N4R and 1407-Gal4 x UAS-ChR2_mCherry; hTau^2N4R^ neuronal cultures. Lysates with PI were prepared in the presence of phosphatase inhibitors (i.e, Halt protease and phosphatase inhibitor cocktail, Thermo Scientific). In this study, western blot membranes were probed with AT8 antibody first. Then, the membrane was stripped and re-probed with HT7 antibody.
